# Daily Affective Experiences across the Lifespan during the COVID-19 Outbreak

**DOI:** 10.1093/geroni/igaa057.3445

**Published:** 2020-12-16

**Authors:** Da Jiang, Shuang Liu

**Affiliations:** Education University of Hong Kong, Hong Kong, China

## Abstract

Objectives Older adults are considered one of the most vulnerable groups to COVID-19. However, previous studies on emotion and aging have found that older adults report better well-being than younger adults in global survey and daily report. To better understand older adults’ well-being during the COVID-19 outbreak, we examined age differences in daily affective experiences in this study. Method A total of participants from mainland China aged 18 to 85 were recruited to participate in the 14-day daily diary study, after a pretest. Their trait affect and demographic information were measured in the pretest. Their daily affect and stress levels were measured in the daily assessments. Results We found that older adults reported a higher level of low arousal positive affect (e.g., calm) and lower levels of high arousal negative affect (HAN; e.g., anxiety), low arousal negative affect (LAN; e.g., dullness), and perceived stress related to COVID-19 in daily life, compared to younger adults. Discussion These results provide initial evidence of daily affective well-being across different age groups in adulthood during the COVID-19 outbreak. Such information is important for developing interventions to promote better well-being during the COVID-19 outbreak.

